# Prognostic and therapeutic significance of phosphorylated STAT3 and protein tyrosine phosphatase-6 in peripheral-T cell lymphoma

**DOI:** 10.1038/s41408-018-0138-8

**Published:** 2018-11-12

**Authors:** Jing Jing Han, Megan O’byrne, Mary J. Stenson, Matthew J. Maurer, Linda E. Wellik, Andrew L. Feldman, Ellen D. McPhail, Thomas E. Witzig, Mamta Gupta

**Affiliations:** 1 Division of Hematology, Department of Internal-Medicine Mayo Clinic, Rochester, MN 55905 USA; 2 Department of Health Sciences Mayo Clinic, Rochester, MN 55905 USA; 3 Department of Laboratory Medicine and Pathology, Mayo Clinic, Rochester, MN 55905 USA; 4 Department of Biochemistry and Molecular Medicine, George Washington University, GW Cancer Center, Washington, DC 20052 USA

## Abstract

Peripheral T cell lymphomas (PTCL) is a heterogenous group of non-Hodgkin lymphoma and many patients remain refractory to the frontline therapy. Identifying new prognostic markers and treatment is an unmet need in PTCL. We analyzed phospho-STAT3 (pSTAT3) expression in a cohort of 169 PTCL tumors and show overall 38% positivity with varied distribution among PTCL subtypes with 27% (16/59) in PTCL-NOS; 29% (11/38) in AITL, 57% (13/28) in ALK-negative ALCL, and 93% in ALK-pos ALCL (14/15), respectively. Correlative analysis indicated an adverse correlation between pSTAT3 and overall survival (OS). PTPN6, a tyrosine phosphatase and potential negative regulator of STAT3 activity, was suppressed in 62% of PTCL-NOS, 42% of AITL, 60% ALK-neg ALCL, and 86% of ALK-pos ALCL. Loss of PTPN6 combined with pSTAT3 positivity predicted an infwere considered significantferior OS in PTCL cases. In vitro treatment of TCL lines with azacytidine (aza), a DNA methyltransferase inhibitor (DNMTi), restored PTPN6 expression and decreased pSTAT3. Combining DNMTi with JAK3 inhibitor resulted in synergistic antitumor activity in SUDHL1 cell line. Overall, our results suggest that PTPN6 and activated STAT3 can be developed as prognostic markers, and the combination of DNMTi and JAK3 inhibitors as a novel treatment for patients with PTCL subtypes.

## Introduction

Peripheral-T cell lymphomas (PTCL) represent approximately 10% of all lymphomas in the United States^1^. PTCL is a heterogeneous disease and has been categorized by the World Health Organization into several subtypes including peripheral TCL-not otherwise specified (PTCL-NOS), angioimmunoblastic TCL (AITL), anaplastic large cell (ALCL), and the predominant subsets of cutaneous TCL (CTCL)^2^. Because of this broad morphological spectrum and immunophenotypic variations among patients, the pathogenesis of PTCLs remains poorly understood. For most subtypes of PTCL, the frontline treatment regimen is typically combination chemotherapy, such as CHOEP (cyclophosphamide, doxorubicin, vincristine, etoposide, and prednisone)^3^, which offers variable success. Recently, the histone deacetylase inhibitors (HDACI) romidepsin and belinostat have been FDA approved for refractory CTCL^4^, however, targeted therapy for the most common PTCL subtypes is still lacking. There is an unmet need for newer targets and treatment options both in upfront and relapsed settings of PTCL and CTCL.

The signal transducer and activator of transcription 3 (STAT3) pathway is considered a therapeutic target for several aggressive cancers, including various solid tumors, leukemia, and diffuse large B cell lymphoma^5,6^. STAT (STAT1, STAT3, and STAT5) transcription factors regulate various biological processes such as the immune response and cell growth^7,8^. STAT3 activation requires phosphorylation of a tyrosine residue through JAKs and TYK2 kinases and constitutive STAT3 activation in tumor cells provides mitogenic and pro-survival signals. However, in vivo activation of STAT3 and its clinical correlation in PTCL subtypes has not been extensively studied. Genetic mutations in STAT3 or its upstream activators JAK1, JAK2, JAK3, or TYK2 responsible for dysregulation of the JAK–STAT pathway have been previously reported^9^. Similarly, recent studies involving a small fraction of PTCL patients have described missense mutations in JAK1, JAK2, JAK3, STAT3, and STAT5B^10–12^. However, the reported mutation frequency does not represent widespread STAT3 activation found in PTCL patients, which highlights the need to identify additional mechanisms of STAT3 deregulation in PTCL subtypes. Tyrosine phosphorylation of STAT3 is dynamically controlled by upstream kinases (JAK1, JAK2 and JAK3 and TYK2) and the tyrosine phosphatases. Consistent with the same notion, the loss of tyrosine phosphatase activity due to missense mutations or deep deletions has been implicated in elevated JAK/STAT signaling in various hematological malignancies including the deletions of the PTPN2 (TC-PTCP) shown in 6% of T-ALL^13^. Beyond STAT3, phosphatase PTP1B (PTPN1) is known to regulate STAT5 (ref. ^14^), TYK2, and JAK2 (ref. ^15^). In the present study, we focused on identifying the prognostic and mechanistic events related to STAT3 activation in PTCL subtypes. Using a cohort of primary tumor tissues from PTCL patient data set we have analyzed the prognostic significance of pSTAT3 and PTPN6 expression for a broad spectrum of PTCLs. Using pharmacological inhibitors of JAK/STAT, DNA methyltransferase, and histone deacetylase (HDAC) we evaluated the potential implications of JAK/STAT signaling in modulating PTCL cellular response.

## Materials and methods

### Patients characteristics

All the patients included in this study were enrolled in the Molecular Epidemiology Resource (MER) of the University of Iowa/Mayo Clinic Lymphoma Specialized Program of Research Excellence (SPORE). This study was conducted on all randomized patients enrolled in the MER/SPORE with confirmed diagnosis and the classification of PTCL. This study was approved by the human subjects Mayo Clinic institutional review board and written informed consent was obtained from all participants for use of their sample and medical record. All pathology was reviewed by a lymphoma hematopathologist to verify the diagnosis and the classification of TCL according to WHO criteria.

### Human T cell lymphoma cell lines

The ALCL cell lines SUDHL1 and SR786 were obtained from DSMZ, Germany and Karpas299 from American Type Culture Collection (ATCC), Manassas, VA. FEPD, ALCL cell line was provided by Dr. Ahmet Dogan. The cutaneous T cell lymphoma cell lines SeAx (Sézary Syndrome), MyLa (mycosis fungoides), and HuT-78 (Sézary Syndrome) were also purchased from ATCC. Cell lines were grown in RPMI 1640 media supplemented with 10% fetal bovine serum and tested for mycoplsma. Human CD3+ T cells were sorted from peripheral blood mononuclear cells isolated from whole blood of healthy donors.

### Antibodies, drugs, and reagents

Antibodies to pSTAT3 (tyrosine 705), pJAK3, JAK3, and PTPN6 were purchased from Cell Signaling Technologies (Beverly, MA, USA) Antibody to β-Actin was purchased from Santa Cruz (Dallas, TX, USA). P-tyrosine (4G10) antibody was purchased from Millipore. The pharmacological inhibitors romidepsin and azacytidine were purchased from Sigma-Aldrich. JAK3 inhibitor WHIP-154 was purchased from Santa Cruz Biotechnology and JAK2 inhibitor ruxolitinib was procured from ChemieTek (Indianapolis, IN).

### Tissue microarrays and immunohistochemistry

Tissue microarrays (TMA) were constructed using triplicate of 0.6-mm cores from the paraffin-embedded tissue blocks. Nonmalignant tonsils (*n* = 10) were used as normal controls. Immunohistochemistry (IHC) was performed using pSTAT3 and PTPN6 antibodies and IgG control in PTCL TMA (*n* = 169) as previously described^6^. Immunostaining for pSTAT3 as well as that of PTPN6 was assessed semi-quantitatively with following criteria: negative: <10% (−); low: 10–30% (+); moderate: 30–80% (++); and high: >80% (+++). 30% cut-off was used for pSTAT3 and PTPN6 postivity.

### Cell survival and proliferation assay

Cell proliferation was assessed by H^3^-thymidine incorporation assay as previously described^16^. Cell survival was assessed by exclusion of Annexin-V-FITC/propidium iodide staining and quantitatively measured by flow cytometry as previously described^17^.

### Immunoprecipitation and western blotting

To the lysates, 5 μg of specific antibodies were added and complexes were allowed to form by incubating with rotation at 4 °C overnight. A 50% slurry (25 μl) of protein A-sepharose or protein G-sepharose was then added and the incubation continued another 24 h. Immunoprecipitates captured with sepharose beads were washed four times with RIPA buffer and analyzed by immunoblotting. Western blotting was performed as previously described using JAK2, JAK3, pJAK3, pSTAT3, STAT3, and PTPN6 antibodies^16^.

### Quantitative reverse transcriptase PCR

Total RNA was isolated from 3 to 5 million cells with the RNeasy Mini Kit (Qiagen) and cDNA was synthesized using Super Script III First-Strand Synthesis Super Mix (18080–400; Invitrogen) following the instruction manual. Quantitative PCR was performed on the CFX96 real-time PCR detection system (Bio-Rad) using the following specific primers and probes. Data analysis was performed using delta-delta-CT method.

PTPN6 forward primer (5′-CACCATCATCCACCTCAAGT-3′);

PTPN6 reverse primer (5′- TCTCACGCACAAGAAACGTC-3′);

PTPN6 probe is 5′-/56-FAM/CGCTGAACT/ZEN/GCTCCGATCCCA/3IABkFQ/-3′

GAPDH forward primer (5′-GAAGGTGAAGGTCGGAGTC-3′);

GAPDH reverse primer (5′-GAAGATGGTGATGGGATTTC-3′);

GAPDH probe used was 5′-/HEX/CAAGCTTCCCGTTCTCAGCC/3IABKFQ/-3′.

### Statistical analysis

Unless stated otherwise, data presented are the mean ± SEM, statistical comparison was performed by Student’s *T*-test. Overall survival (OS) was defined as the time sample biopsy to death of any cause. Patients without an event were censored at last known follow-up. Combination index (CI) was analyzed using the Chou–Talalay equation^18^. *P-*values ≤0.05 were considered significant.

## Results

### In vivo STAT3 activation in PTCL subtypes

**Fig. 1:**
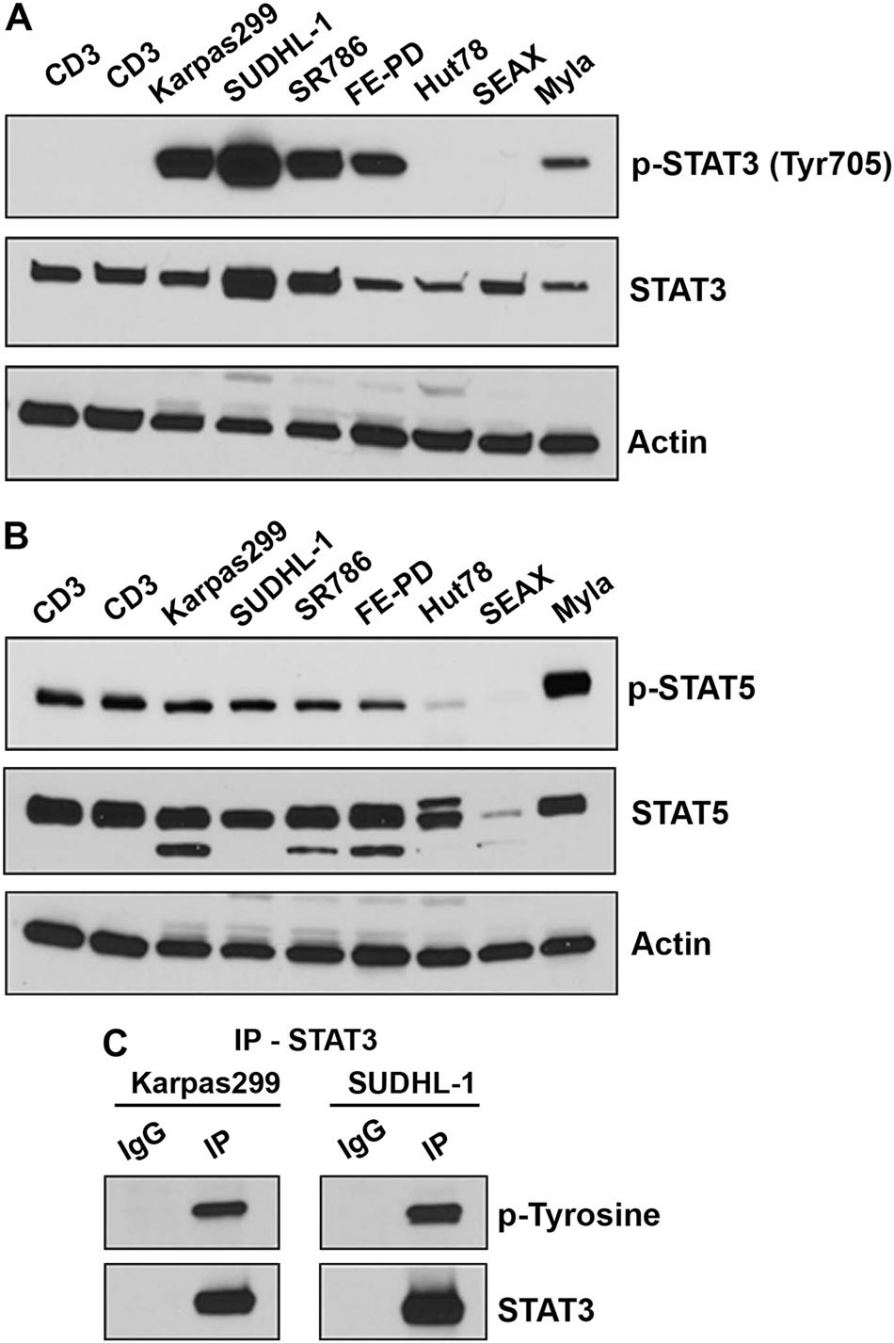
STAT3 tyrosine activation in human TCL cell lines. (**A**, **B**) Images of western blots showing STAT3 (**A**) or STAT5 (**B**) tyrosine phosphorylation in untreated TCL cell lines and normal CD3^+^ T cells (*n* = 3) sorted from whole blood of healthy donors, and (**C**) immunoblot showing label of phospho-tyrosine and STAT3 in protein lysates after immunoprecipitation (IP) with IgG or STAT3 antibodies

To detect pSTAT3 in PTCL subtypes we performed IHC on the TMA from pretreatment tumor biopsies collected from 169 PTCL patients. Using a cut-off of ≥30% (≥++) pSTAT3 staining 38% (64/169) PTCL tumors were pSTAT3 positive (Supplementary Figure 1). pSTAT3 positivity in major subtypes PTCL-NOS, AITL, ALK-neg ALCL, and ALK-pos ALCL TCL was 27%, 29%, 57%, and 93%, respectively (Table 1a). Interestingly, pSTAT3 positivity was also detected in certain rare PTCL subtypes such as NK/T cell (6/12; 50%) and enteropathy type (2/3; 33%) while pSTAT3 in some of rare subtypes was either low (17% 1/6) in cutaneous ALCL or totally absent in mycosis fungoides (MF, 0/5 positive) samples analyzed (Table 1b). These results suggest that pSTAT3 is differentially expressed among PTCL subtypes. Consistent with these results from IHC, constitutive pSTAT3 expression was also detected in TCL cell lines by western blotting (Fig. 1a). While pSTAT3 expression was robust in all three ALCL cell lines (Karpas299, SUDHL1, and SR786), only moderate pSTAT3 signal was detected in MF origin CTCL lines (MyLa); CTCL lines of SS origin (Hut-78 and SeAX) like normal CD3+ T cells lacked pSTAT3 expression. Due to the lack of the human derived PTCL-NOS and AITL cell lines, we were unable to perform pSTAT3 activation in these PTCL subtypes.

**Table 1a:** Analysis of pSTAT3 expression in untreated PTCL tumor biopsies (*n* = 169)

(A)^a^			
PTCL major subtypes	Total	*N* pSTAT3+	% pSTAT3+ (30% cut-off)
PTCL, NOS	59	16	27
Angioimmunoblastic	38	11	29
ALCL (ALK negative)	23	13	57
ALCL (ALK positive)	15	14	93

(**A**) pSTAT3 was done on the TMA from (*n* = 59), AITL (*n* = 38), and ALCL negative (*n* = 23) and ALCL positive (*n* = 15) patients by immunohistochemistry (IHC).

**Table 1b:** ▓▓

(B)^b^			
Other TCL subtypes	Total (*n*)	pSTAT3+ (*n*)	pSTAT3+ (%)
NK/T cell	12	6	50
Enteropathy type	3	1	33
Hepatosplenic	2	0	0
Subcutaneous panniculitis-like	1	1	100
Cutaneous ALCL	6	1	17
Mycosis fungoides (MF)	5	0	0
Sezary syndrome (SS)	None		
Large granular lymphocytic leukemia (LGL)	5	1	20

(**B**) pSTAT3 staining was done in rare TCL subtypes by IHC.

Beyond STAT3, STAT5 is another major component in the JAK/STAT pathway. Interestingly, unlike pSTAT3, expression of pSTAT5 in all TCL lines tested was comparable to that in CD3+ normal T cells, only exception was Myla cell line of MF origin, which showed up-pSTAT5 as compared to normal CD3 cells **(**Fig. 1b). To further confirm that the phosphorylation was specific to STAT3, STAT3 proteins were pulled down and immunoblotted to detect phospho-tyrosine (Fig. 1c). This analysis revealed that tyrosine phosphorylation detected in TCL cell lines is primarily on STAT3. Taken together, these results suggest that constitutive pSTAT3 expression is highly frequent across TCL subtypes and that the STAT3 phosphorylation occurs regardless of ALK expression status.

### STAT3 phosphorylation and prognosis in PTCL subtypes

**Fig. 2:**
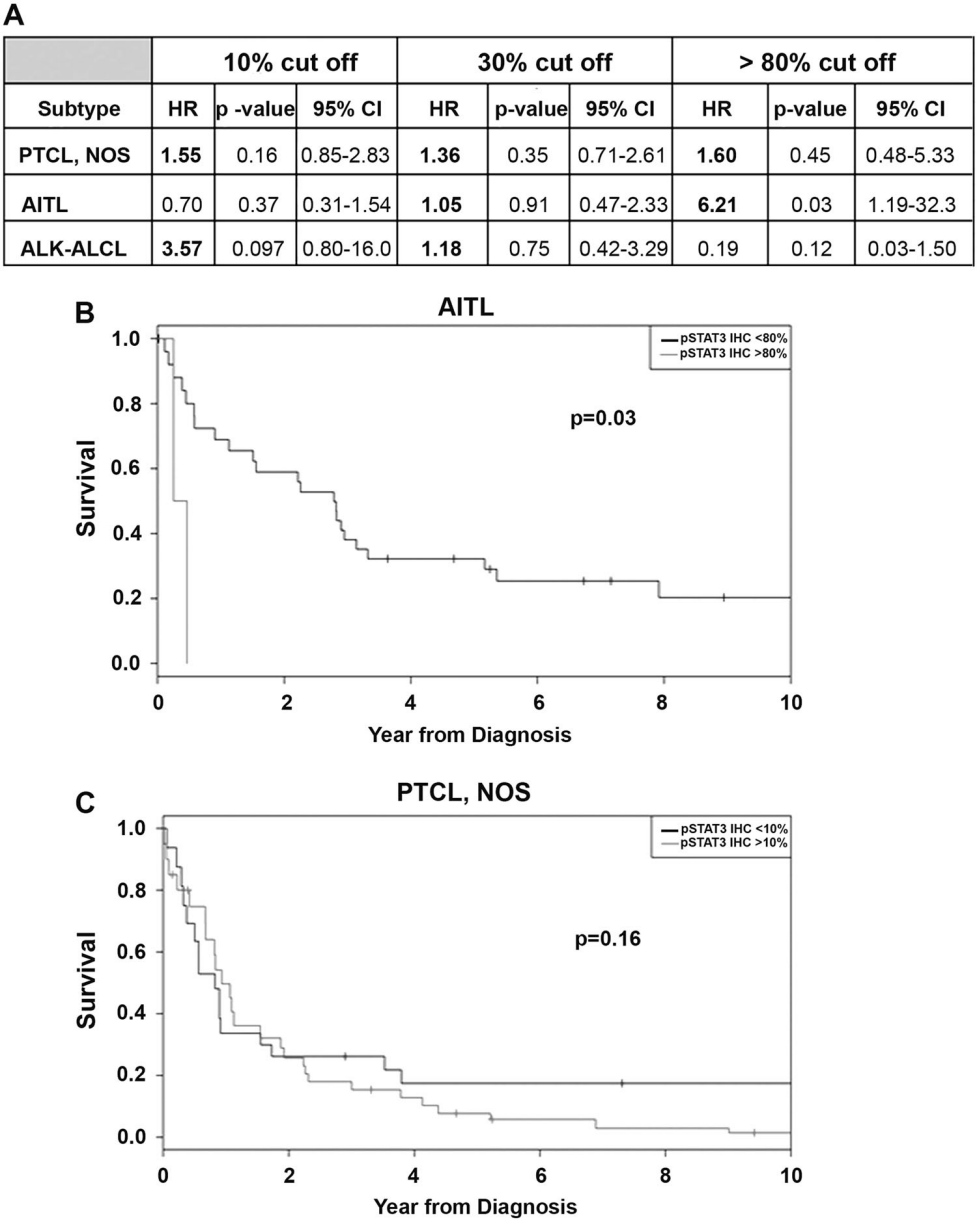
Correlation of pSTAT3 expression label at diagnosis to overall survival in PTCL patients treated with standard chemotherapy. (**A**) Hazard ratio with 10%, 30%, and 80% cut-off pSTAT3 positivity in PTCL-NOS, AITL, ALK-neg ALCL, and ALK-pos ALCL. (**B**, **C**) Kaplan–Meier plots of the OS of AITL (**B**) and PTCL-NOS (**C**) patients with differential pSTAT3 positivity

The association between pSTAT3 positivity and OS was evaluated using the 10%, 30% or 80% cut-off for PTCL-NOS, AITL, and ALK-negative ALCLs cohorts of PTCL patients (Fig. 2a). Hazard ratio (HR) with 30% cut-off for pSTAT3 was >1.0 for PTCL-NOS (HR = 1.36, *p* = 0.35), AITL (HR = 1.05, *p* = 0.91), and ALK negative ALCL (HR = 1.18, *p* = 0.75), respectively (Fig. 2a). Interestingly, with 80% cut-off (showing strong pSTAT3 positivity), the correlation was highly significant for the AITL subgroup (HR = 6.2; *p* = 0.03) but not for the PTCL-NOS (HR = 1.6, *p* = 0.45) (Fig. 2a). Kaplan–Meier (KM) survival graphs indicate that pSTAT3 positivity was marginally associated with inferior OS in AITL (80% cut-off) and PTCL-NOS patients (10% cut-off) (Fig. 2b, c). Due to the small sample number in the ALK-negative subgroup we were unable to perform OS analysis in this subgroup. These results indicate that pSTAT3 only weakly correlates with OS in some PTCL subtypes.

### Mechanisms of STAT3 dysregulation in PTCL subtypes

STAT3 phosphorylation leading to its activation is regulated through several distinct mechanisms. We have shown that serum cytokines including sIL-2Rα and IL-1Rα were overexpressed in the PTCL patients and correlated with poor prognosis^19^. Commonly known mechanisms of STAT3 activation involve missense mutations in genes encoding STAT3 itself or upstream activators such as JAK1–3, loss of tyrosine phosphatases, which activate canonical JAK/STAT signaling^20^. We focused in detail on understanding the regulation of pSTAT3 via negative feedback mechanisms involving the protein tyrosine phosphatases involved in de-phosphorylation of STAT3.

### *Protein tyrosine phosphatase* PTPN6 suppression and clinical correlation in PTCL subtypes

**Fig. 3:**
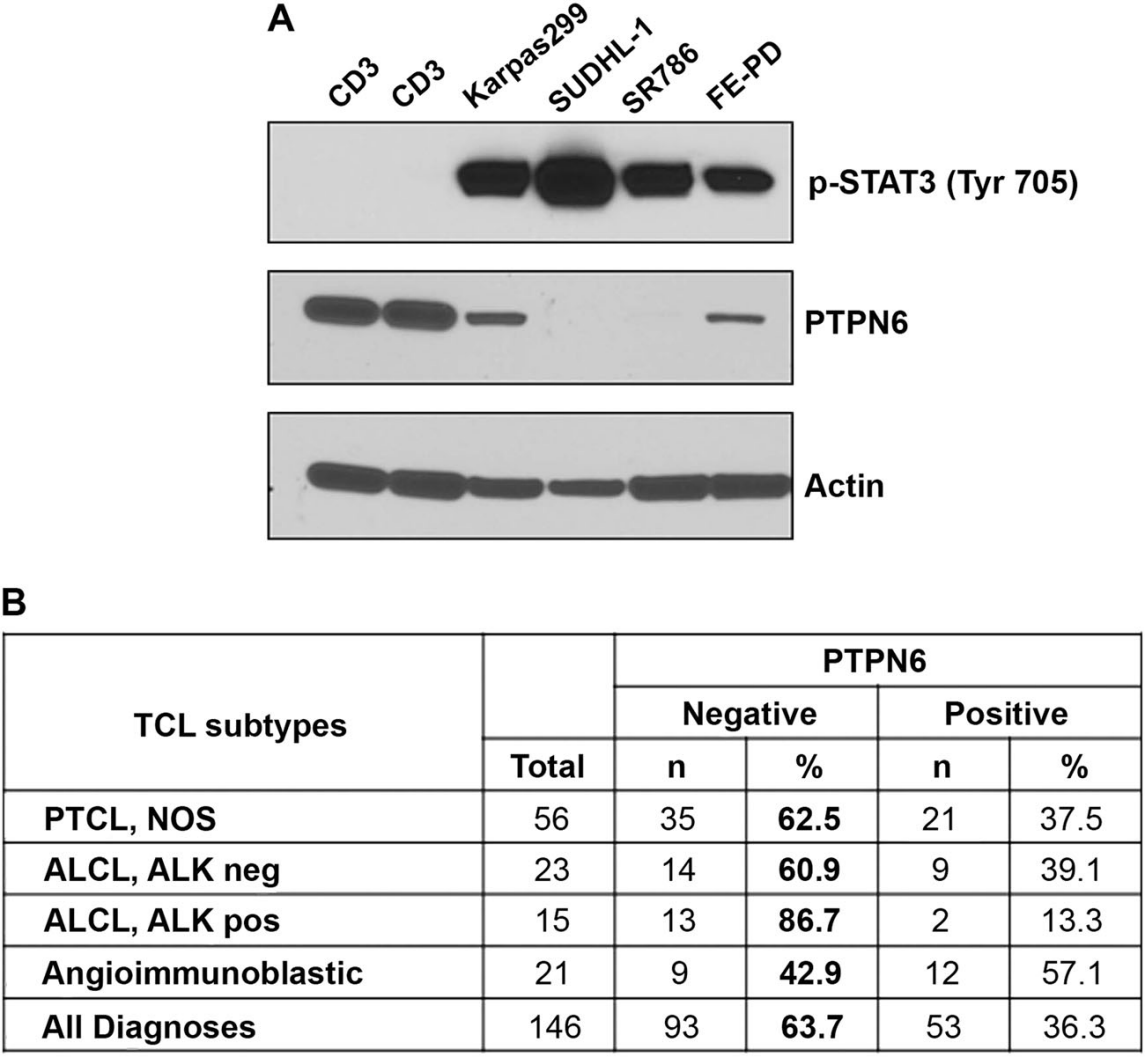
PTPN6 expression in TCL cell lines and patient tumor specimens. (**A**) A representative western blots showing pSTAT3 and PTPN6 expression in TCL cell lines (*n* = 4) and CD3^+^ normal T cells (*n* = 2) sorted from blood of healthy donors; β-actin was used as a loading control. (**B**) Detection of PTPN6 on untreated PTCL-NOS (*n* = 56), ALCL ALK negative (*n* = 23), ALCL ALK positive (*n* = 15), and AITL (*n* = 21) tumors specimens by IHC. Thirty percent cut-off was used for PTPN6 postivity

First, we examined the expression of tyrosine phosphatases PTP1B, PTPN2, and PTPN6 in TCL cell lines Karpas 99, SUDHL1, SR786 and FEPD and normal CD4^+^ T cells by western blotting. PTPN6 was suppressed in Karpas299 and FEPD cells; however, PTPN6 was silenced in SUDHL1 and SR786 cells as compared to normal CD4^+^ T cells (Fig. 3a). Expression of PTP1B and PTPN2 was undetectable by western blotting (data not shown) in TCL lines. Interestingly, loss of PTPN6 expression was inversely correlated with pSTAT3 expression, with increased pSTAT3 in TCL cell lines lacking PTPN6 as opposed to the lack of pSTAT3 in normal T cells with high PTPN6 expression (Fig. 3a). This in vitro analysis indicated that PTPN6 could be a negative regulator of STAT3 activation in TCL lines of ALCL origin. Due to lack of the PTCL-NOS and AITL subtypes human-derived cell lines, we were unable to identify the PTPN6 and pSTAT3 correlation in these subtypes. We next examined PTPN6 expression in vivo using TMA made from untreated PTCL patients (*n* = 169). IHC analysis showed PTPN6 negativity in 63.7% (93/147) of all PTCL patient tissues with 62% (35/56) of PTCL-NOS, 60% (14/23) of ALK-neg ALCL, 86% (13/15) of ALK-pos ALCL, and 42% (9/21) of AITL analyzed were PTPN6 negative (Fig. 3b). These results suggest that PTPN6 is silenced in more than 40–60% of the PTCL-NOS, ALK-neg ALCL, AITL, and 86% in ALK-pos ALCL subtypes, and may be an important regulatory mechanism of STAT3 activation in PTCL patients.

**Fig. 4:**
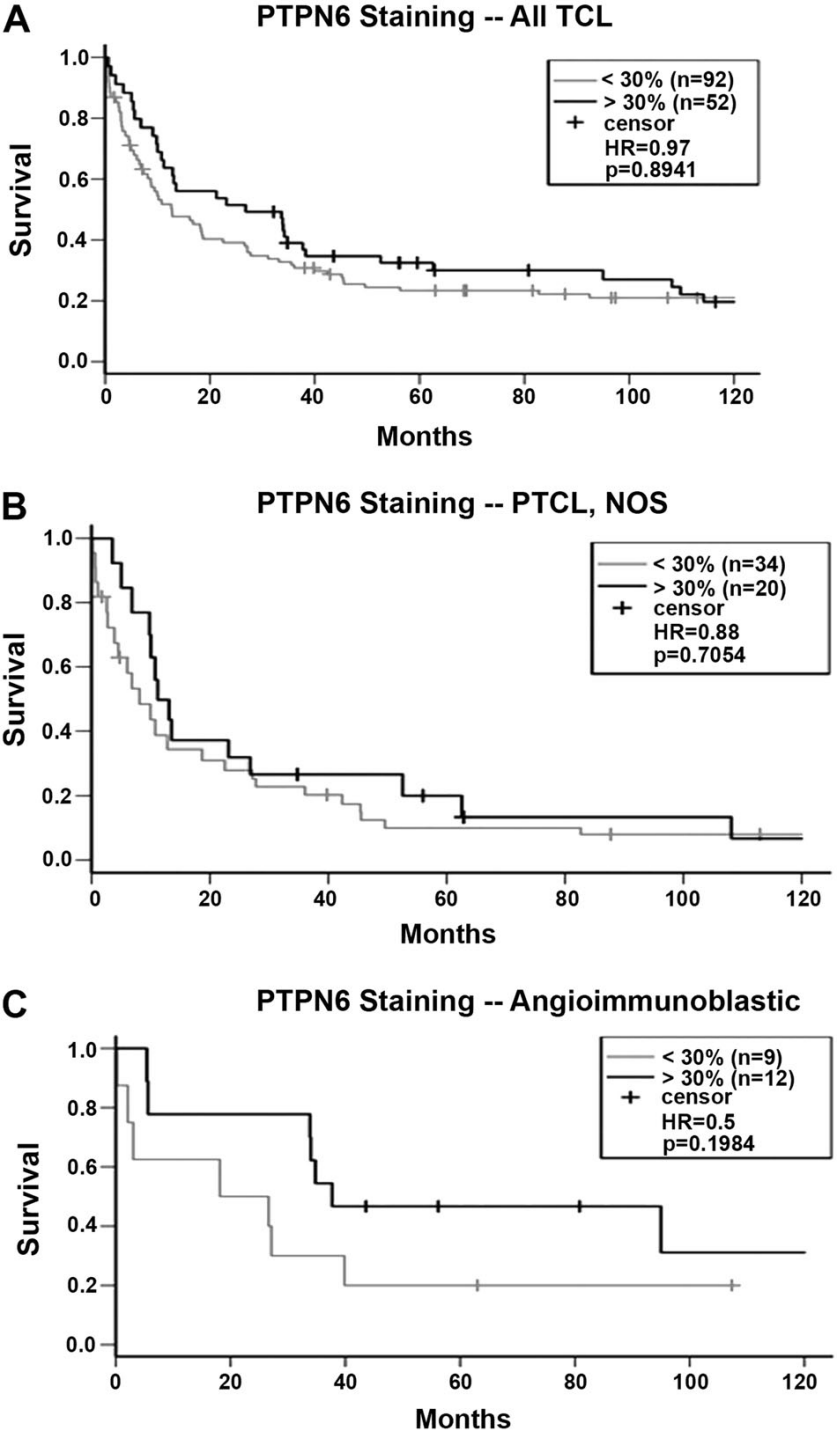
PTPN6 expression and prognosis within PTCL subtypes. (**A**) Overall survival (OS) by PTPN6 expression in all PTCL tumors (*n* = 144). (**B**) OS by PTPN6 positivity in PTCL-NOS tumors (*n* = 54). (**C**) OS by PTPN6 positivity in AITL patients (*n* = 21)

**Fig. 5:**
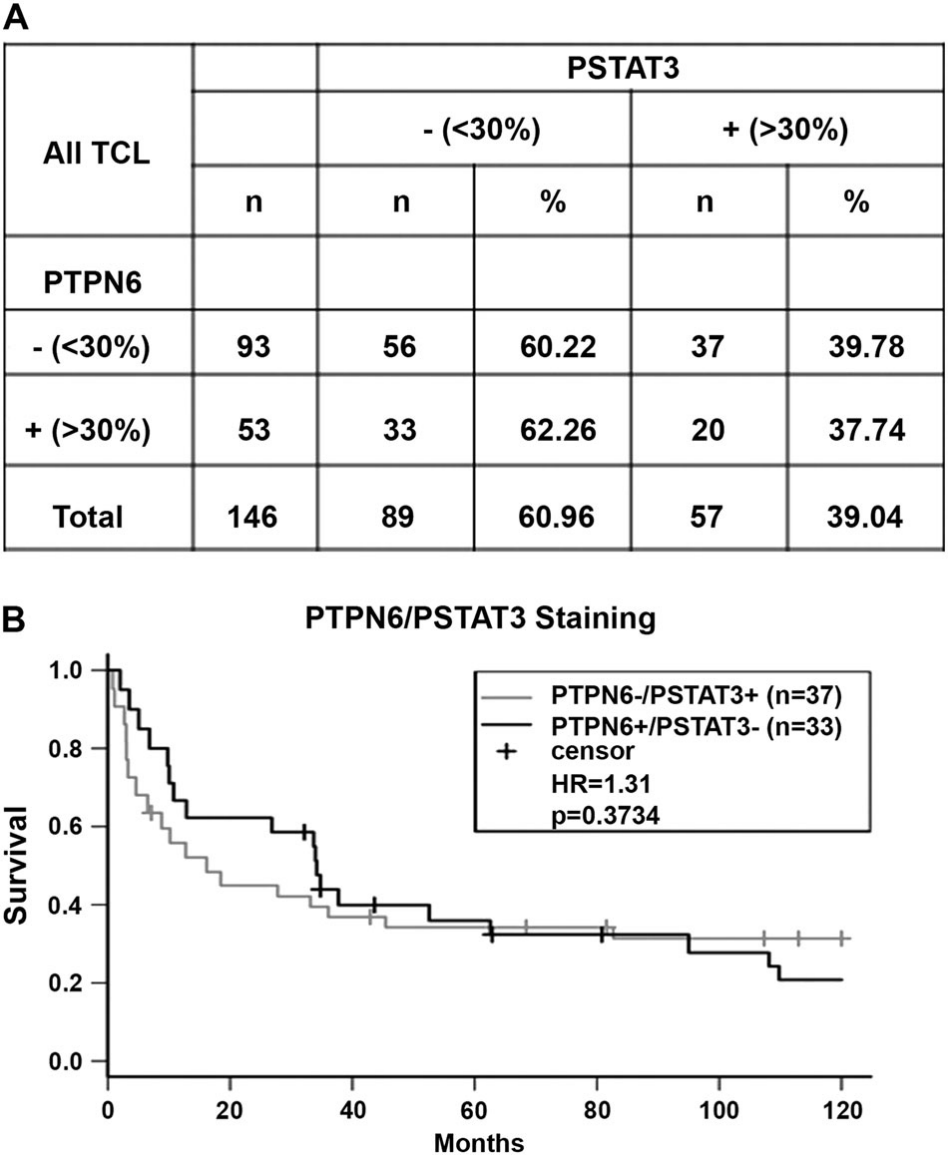
Correlation of PTCL tumor pSTAT3 and PTPN6 expression. (**A**) Correlation of PTPN6 expression with pSTAT3 by IHC in PTCL patients (*n* = 146). (**B**) Survival analysis for PTPN6 negative/pSTAT3+ (*n* = 37) and PTPN6+/pSTAT3 (*n* = 33) negative group was done by KM graphs

We next evaluated the association between PTPN6 expression and OS in PTCL patients. As shown in KM survival graphs, low PTPN6 expression correlates with inferior OS not only in all PTCL patients all together (HR = 0.97, *p* = 0.89), but also when PTCL-NOS and AITL patient cohorts were analyzed separately (Fig. 4a–c). Although, this data did not reach statistical significance, this was likely due to the small sample size in PTPN6 positive and negative groups. Negative correlation of STAT3 and PTPN6 in PTCL tumor samples was also analyzed by IHC (*n* = 146). Our results showed that 37 out of 93 PTPN6-negative specimens were positive for pSTAT3 (39.7%), while 20 out of 53 PTPN6 positive cases were pSTAT3 positive (37.8%) (Fig. 5a). Overall, these results suggest a trend of negative correlation between PTPN6 and pSTAT3 in PTCL tumor samples. Correlative analysis in pSTAT3-positive PTCL patients grouped by differential PTPN6 expression showed worse OS in PTPN6 low/pSTAT3+ (*n* = 37) compared to the PTPN6-High/pSTAT3+ (*n* = 33) group (HR = 1.3, *p* = 0.37; Fig. 5b). Although statistically insignificant, these results suggest that lack of PTPN6 expression influences the prognostic effect of STAT3 activation in PTCL patients.

Silencing of *PTPN6* gene expression in cancer cells due to DNA methylation in promiter via DNA methyltransferases 1 (DNMT1) has been widely described^21–23^. Similarly, drugs targeting DNMTs or HDACs are also known to induce re-expression of silenced genes via various mechansims. We sought to determine the effect of the DNMT inhibitor azacytadine (aza) and FDA approved HDAC inhibitor romidepsin (rom) on the expression of *PTPN6* in SUDHL1 cells. While the HDAC inhibitor romidepsin had no significant change in PTPN6 expression, treatment with 1 µM aza enhanced PTPN6 mRNA transcript level by 1.7-fold relative to the corresponding vehicle control in SUDHL1 cells (Supplementary Figure 2; S2). These results suggest that DNMT mediated promoter methylation is a primary cause of PTPN6 downregulation in PTCL cells.

### Constitutive activation of JAK1-3 kinases in TCL cell lines

**Fig. 6:**
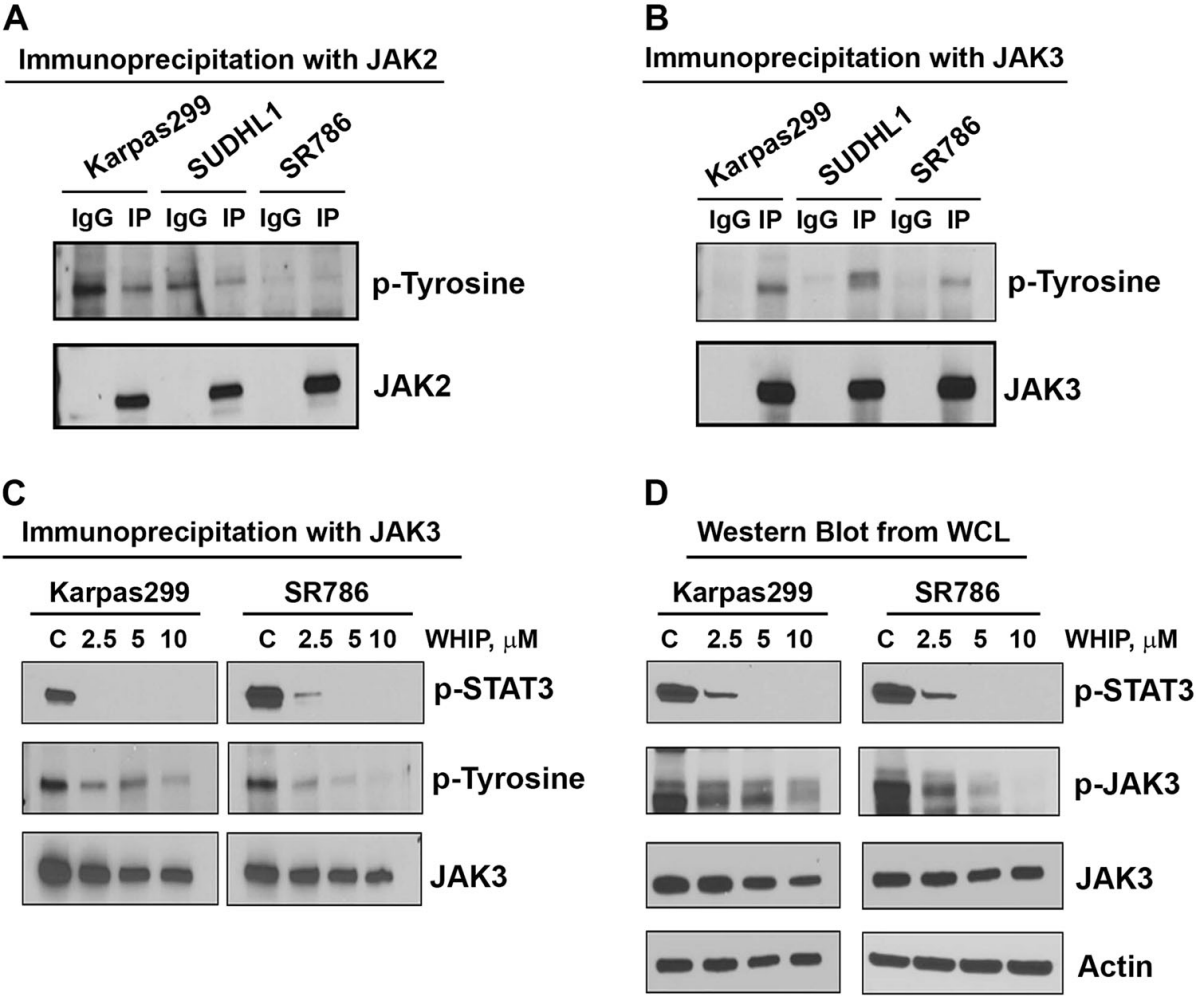
JAK2 and JAK3 Kinases activation in TCL cell lines. (**A**, **B**) JAK2 or JAK3 was immunoprecipitated from lysates of three TCL cell lines and the immuno-complex was examined for phospho-tyrosine antibody. (**C**) After a 24-h treatment of Karpas299 and SR786 cell lines with JAK3 inhibitor WHIP-154, JAK3 immunoprecipitates were probed with phospho-tyrosine and pSTAT3 antibodies. (**D**) Karpas299 and SR786 cells were treated with various concentrations of WHIP-154 for 18-h and and then blotted with pSTAT3 and pJAK3 antibodies

The constitutive activation of STAT3 signaling pathways suggested the deregulation of upstream activators, including the JAK family of proteins. Next we explored that whether STAT3 upstream regulators JAK2 or JAK3 kinase is responsible for the STAT3 activation in TCL. There was no detectable phosphorylation in immunoprecipitates of JAK2 antibody in any of the three cell lines tested as compared with IgG control (Fig. 6a). A robust phosphorylation was detected by tyrosine antibody in the immunoprecipites of JAK3 antibody as compared to IgG control from the Karpas299, SUDHL1, and SR786 cell lines (Fig. 6b). These results suggest that JAK3 but not JAK2 is responsible for the STAT3 phosphorylation in TCL. To further confirm the role of JAK3 in deregulation of STAT3 signaling we determined the effect of pharmacological inhibitor WHIP-154 on tyrosine activity of STAT3. After treatment of Karpas299 and SR786 cell lines with JAK3 inhibitor WHIP-154, JAK3 immunoprecipitates were probed with phospho-tyrosine and pSTAT3 antibodies. As shown in Fig. 6c, treatment with WHIP-154 attenuated pSTAT3 and p-tyrosine levels in both the cell lines tested. Furthermore, inhibition of pSTAT3 and pJAK3 was also detected in whole-cell lysates of Karpas299 and SR786 cells by western blotting (Fig. 6d). These results suggest that indeed JAK3 plays an important role in STAT3 activation in PTCL subtypes.

### Sensitization of TCL cells to JAK3 inhibitor by DNMT inhibition

We have shown above that constitutive activation of STAT3 signaling in TCL cells can be targeted through upstream kinase JAK3. However, the efficacy of JAK3 or JAK1/JAK2 inhibitors can be limited by loss of PTPN6, which augments STAT3 activation in PTCL cells. We therefore examined if DNMT inhibition by aza leading to enhanced PTPN6 expression could suppress pSTAT3 in TCL cells and whether re-expression of PTPN6 by DNMT inhibitor improves antitumor effects of JAK/STAT inhibitors.

**Fig. 7:**
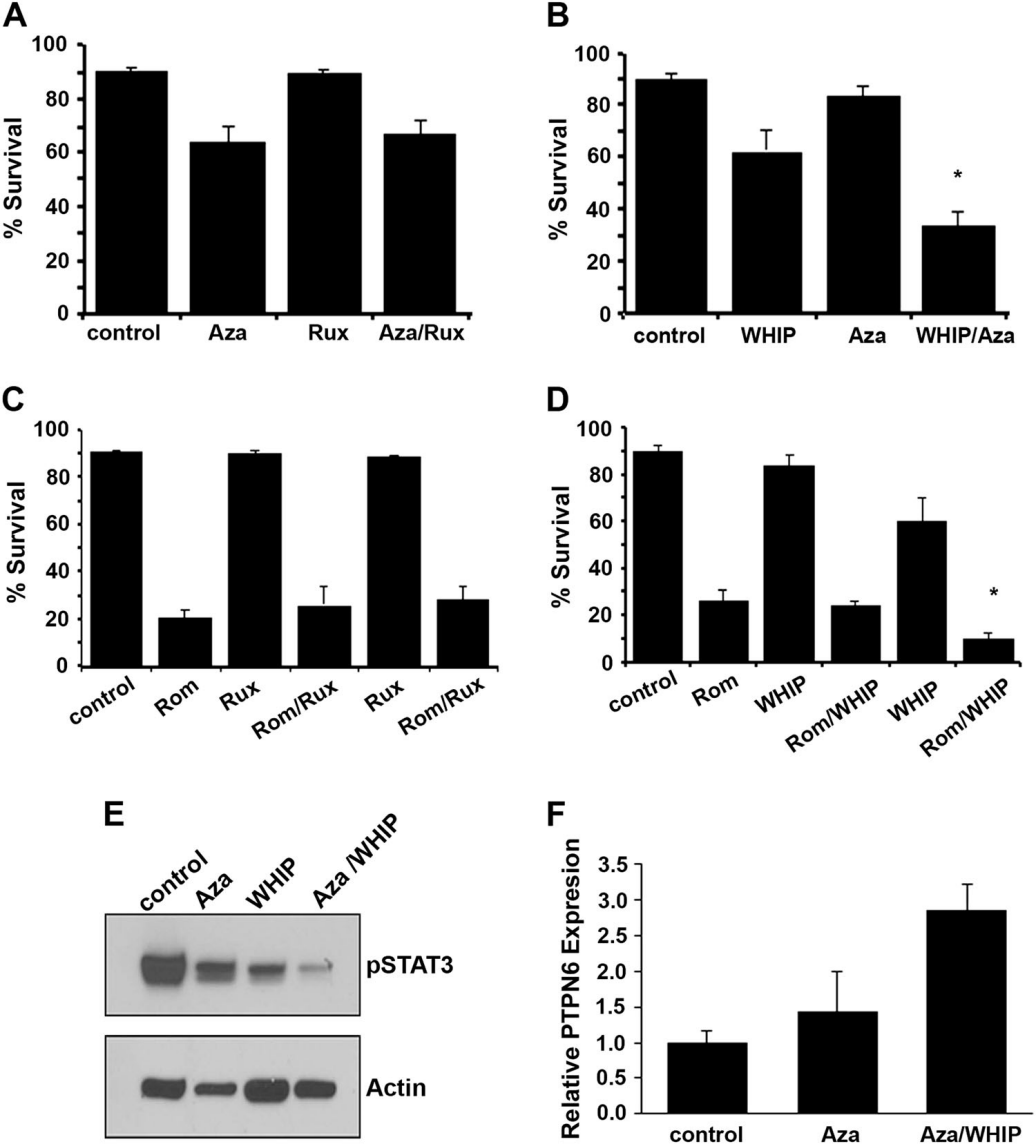
Co-treatment with epigenetic inhibitor with JAK/STAT pathway inhibitors in SUDHL1 cells. (**A**, **B**) SUDHL1 cells were treated with the indicated concentrations of (**A**) azacytidine (2.5 µM) ± ruxolitinib (2.5 µM), (**B**) azacytidine (2.5 µM) ± WHIP-154 (2.5 µM) and cells were treated 48 h and survival was assessed as described. (**C**, **D**) SUDHL1 cells were treated with (**C**) romidepsin (2.5 nM) + ruxolitinib (2.5 or 5.0 µM) (**D**), romidepsin (2.5 nM) + WHIP-154 (2.5 and 5.0 µM) for 48 h and survival was assessed as described before. Presented in the bar graphs are mean ± standard error of three independent experiments. *P-value≤0.05 was considered significant

Cytotoxicity of dual JAK1/JAK2 inhibitor ruxolitinib (RUX) and JAK3 inhibitor WHIP-154 (WHIP) along with DNMT inhibitor azacytidine (aza) as a single agent or in combination was assessed in SUDHL1 cell line (Fig. 7a–d). JAK1/JAK2 inhibitor RUX in combination with aza had no significant effect on cell survival as compared to drug alone (Fig. 7a). Combining JAK3 inhibitor WHIP with aza had a robust 2–3-fold decrease in cell survival as compared to either drug alone (*p* = 0.02) (Fig. 7b). CI using the Chou–Talalay equation^18^ indicated that aza and WHIP-154 were synergistic, as indicated by CI value < 1.0 (i.e., CI was 0.6 at 5 nM). The class I HDAC inhibitor romidepsin (FDA approved for relapsed PTCL) alone was highly effective as evident by 80% decrease in cell survival as compared to control. Interestingly, combining romidepsin with dual JAK1/JAK2 inhibitor Rux had no further advantage (Fig. 7c). Conversely, combined treatment of JAK3 inhibitor WHIP (2.5 µM) further enhanced antitumor efficacy of romidepsin in SUDHL1 cells (*p* = 0.001)(Fig. 7d).

We then sought to examine the effect of JAK3 and DNMT inhibition through WHIP and aza on pSTAT3 expression in TCL cells. The effect of WHIP, aza, or their combination on pSTAT3 was determined by western blotting in SUDHL1 cells. As shown in Fig. 7e, treatment of SUDHL1 cells with 2.5 µM WHIP or 5 µM aza used alone decreases pSTAT3 level, but a combination of WHIP and aza had a more robust inhibition of pSTAT3 levels as compared to either drug alone, while the there was no effect on the expression of the housekeeping gene β-actin (Fig. 7e). Furthermore, the combination of WHIP with aza led to a marked increase in PTPN6 expression as compared to control or treatment with either drug alone (Fig. 7f). Collectively, these data suggest that DNMT inhibitor can increase PTPN6 expression and sensitize PTCL cells to JAK3 inhibitors.

## Discussion

Constitutively active STAT3 signaling promotes tumor growth by upregulation of genes involved in cell survival^24^. Prior studies from our laboratory have reported that constitutively expressed pSTAT3 is indeed responsible for promoting malignant behavior in DLBCL^6,25^. Similarly, several prior reports describe the role of STAT3 in ALK + ALCL; however, there is a lack of data relating pSTAT3 to survival parameters in other major PTCL subtypes. Here we investigated pSTAT3 expression by IHC in primary tumor samples from 169 patients samples and found that 27% PTCL-NOS, 29% AITL, and 57% ALCL ALK-negative tumors express pSTAT3. Our retrospective analysis of correlation indicate marginal association between pSTAT3 and OS in PTCL-NOS and AITL tumors (HR ratio >1.0). A number of studies have reported STAT3 activation through tyrosine phosphatase PTPN6 in hematological malignancies^26,27^. Our results suggest that PTPN6 deficiency (assessed by IHC) occurs in 63% of all TCL tumors equally distributed across various TCL subtypes (62% PTCL-NOS, 42% AITL, and 60% ALK negative ALCL). Patients with tumors with suppressed or absent PTPN6 tended to do somewhat worse although again this was not statistically significant and lack of PTPN6 expression appears to adversely correlate with OS. Furthermore, suggesting a link with STAT3 dysregulation, 62% of PTPN6 positive PTCLs analyzed were negative for pSTAT3 expression. Retrospectively analyzed, these TCL patients (PTPN6 positive and pSTAT3 negative) had better OS than other counterparts indicating that the PTPN6–pSTAT3 axis is biologically important to aggressive behavior in PTCL.

Epigenetic inhibitors such as HDAC and DNA methyltransferase (DNMT) inhibitors are known to activate silenced genes in cancer via various mechansims^26,28–31^. Our results demonstrate that the DNMT inhibition upregulates PTPN6 expression in TCL cells. This result are consistent with the previously known epigenetic silencing process associated with the promoter hypermethylation on certain tumor suppressor genes including PTPN6 (ref. ^23^). It is well documented that DNA hypermethylation is catalyzed by the DNA methyltransferase family of enzymes, including DNMT1, DNMT3a, and DNMT3b^32^, we speculate that the mechansims underlying the PTPN6 re-expression involves DNMTi mediated reversion of CPG hypermethylation in promoter region of PTPN6. Several JAK/STAT pathway inhibitors are in clinical trials or approved for myeloproliferative disorders^33,34^. We and others have previously reported JAK2 inhibition is highly cytotoxic to pSTAT3 positive DLBCL cells^35–37^. Our finding that JAK3 kinase is constitutively activated in the TCL cells and is responsible for STAT3 activation is consistent with prior studies^12,38^.

Beyond JAK3-mediated STAT3 phosphorylation, epigenetic silencing of negative regulators such as PTPN6 may contribute towards sustained STAT3 phosphorylation in cancer cells. Therefore, PTPN6 re-expression by DNMTi may augment effects of JAK3 inhibition on STAT3 phosphorylation in PTCL. Indeed, combining azacytidine with JAK3 inhibitor showed a synergistic inhibition of TCL cell survival. The detailed mechansims underlying the regulation of JAK3 by DNMTs remain to be determined. Azacytidine has been tested in clinical trial studies and dose-limiting toxicities have been a major obstacle in translating DNMT inhibitors when used alone^32,39^. Whether combining azacytidine with JAK3 inhibitors improves tolerability and efficacy in PTCL remains to be seen, although pSTAT3 targeting by combined inhibition of JAK3 and DNMT seems promising. While the FDA-approved HDAC inhibitors produce prolonged responses in some patients, the overall response rate in CTCL and PTCL is relatively low (~30%), highlighting the need for more effective treatments^40^. Rationally designed studies in which HDAC inhibitors are combined with other novel agents are being actively pursued^40^. Several ongoing clinical trial studies are examining various HDAC inhibitors with chemotherapy regimens^41,42^ or other novel agents^43,44^ for the treatment of TCL. Given the robust pSTAT3 expression in PTCLs, combining HDAC inhibitors with inhibitors of JAK/STAT pathways can be an effective strategy to achieve durable remission.

In conclusion, these data provide a comprehensive characterization of the epigenetic mechanisms leading to STAT3 dysregulation in PTCL tumors including PTCL-NOS and AITL. These findings support the notion that control of pSTAT3 in TCL tumors is complex and involves epigenetic silencing of the tyrosine phosphatase PTPN6. The presence of PTPN6 expression with the lack of pSTAT3 expression can be a better prognostic biomarker than pSTAT3 alone. These findings also identify a unique combination of JAK3 and DNMT inhibitors to treat pSTAT3-bearing PTCL patients. Therefore, this study clearly provides a rationale to launch the study of pSTAT3 and PTPN6 by IHC in large prospective trials of PTCLs and to conduct in vivo evaluation of epigenetic inhibitors combined with specific JAK3 inhibitors.

## Electronic supplementary material

S1

S2
