# Supplementary figures and images for: Prognostic and therapeutic significance of phosphorylated STAT3 and protein tyrosine phosphatase-6 in peripheral-T cell lymphoma
Source: Blood Cancer J. 2018 Nov 12;8(11):110. doi: 10.1038/s41408-018-0138-8 (PMC6232096; doi:10.1038/s41408-018-0138-8)

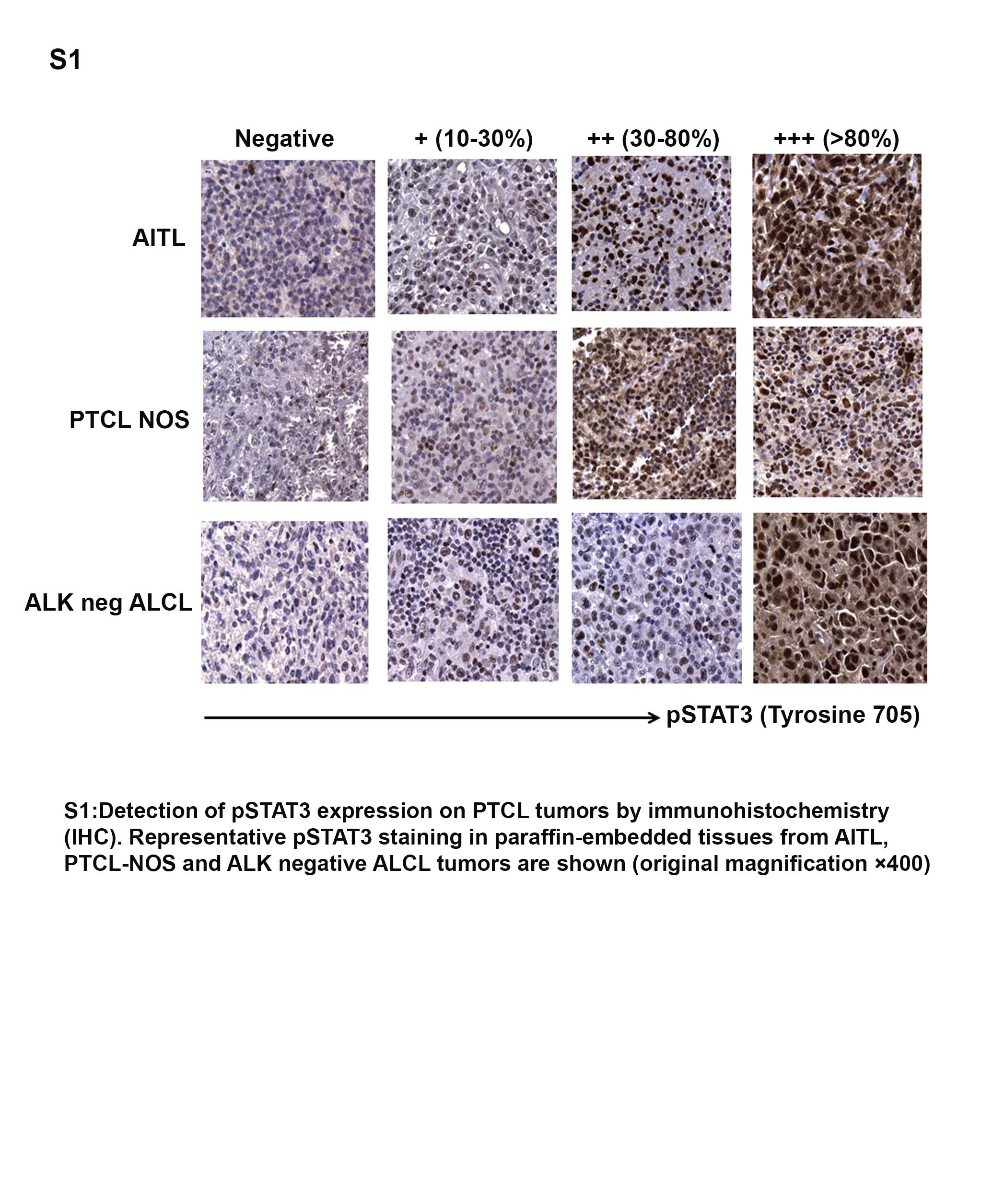

Supplement: Supplementary file 1 — S1 [file 41408_2018_138_MOESM1_ESM.jpg]

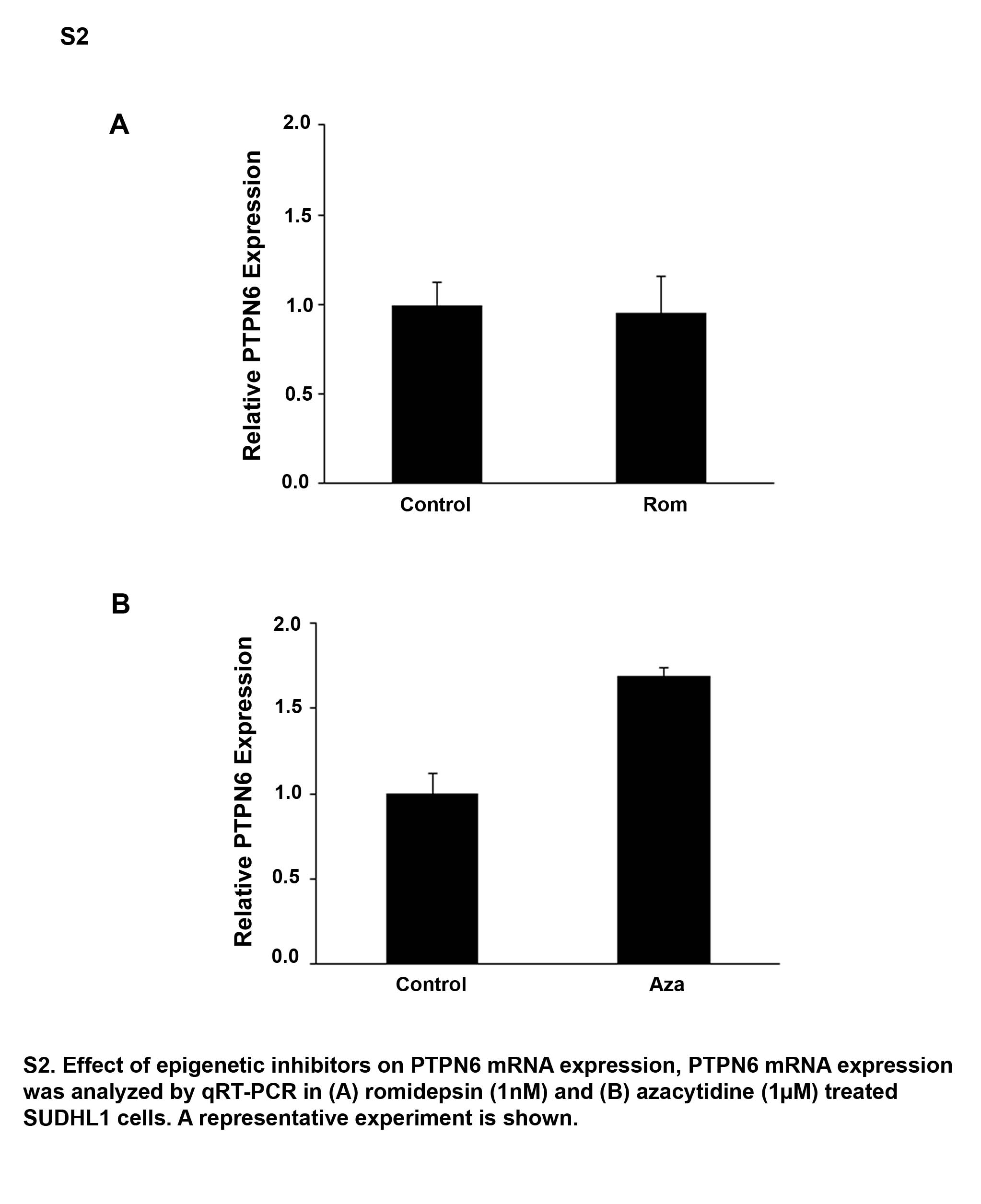

Supplement: Supplementary file 2 — S2 [file 41408_2018_138_MOESM2_ESM.jpg]
